# Racing and Pacing in the Reward System: A Multi-Clock Circadian Control Over Dopaminergic Signalling

**DOI:** 10.3389/fphys.2022.932378

**Published:** 2022-06-23

**Authors:** Kamil Pradel, Gniewosz Drwięga, Lukasz Chrobok, Tomasz Błasiak

**Affiliations:** ^1^ Department of Neurophysiology and Chronobiology, Institute of Zoology and Biomedical Research, Jagiellonian University, Kraków, Poland; ^2^ School of Physiology, Pharmacology and Neuroscience, University of Bristol, University Walk, Bristol, United Kingdom

**Keywords:** dopamine, extra-SCN oscillators, circadian clock, ventral tegmental area, substantia nigra pars compacta, timescale, dopaminergic system, multi-clock model

## Abstract

Level of motivation, responsiveness to rewards and punishment, invigoration of exploratory behaviours, and motor performance are subject to daily fluctuations that emerge from circadian rhythms in neuronal activity of the midbrain’s dopaminergic system. While endogenous circadian rhythms are weak in the ventral tegmental area and substantia nigra pars compacta, daily changes in expression of core clock genes, ion channels, neurotransmitter receptors, dopamine-synthesising enzymes, and dopamine transporters, accompanied by changes in electrical activity, are readily observed in these nuclei. These processes cause dopamine levels released in structures innervated by midbrain dopaminergic neurons (e.g., the striatum) to oscillate in a circadian fashion. Additionally, growing evidence show that the master circadian clock located in the suprachiasmatic nucleus of the hypothalamus (SCN) rhythmically influences the activity of the dopaminergic system through various intermediate targets. Thus, circadian changes in the activity of the dopaminergic system and concomitant dopamine release observed on a daily scale are likely to be generated both intrinsically and entrained by the master clock. Previous studies have shown that the information about the value and salience of stimuli perceived by the animal is encoded in the neuronal activity of brain structures innervating midbrain dopaminergic centres. Some of these structures themselves are relatively autonomous oscillators, while others exhibit a weak endogenous circadian rhythm synchronised by the SCN. Here, we place the dopaminergic system as a hub in the extensive network of extra-SCN circadian oscillators and discuss the possible consequences of its daily entrainment for animal physiology and behaviour.

## Introduction

Historically, the suprachiasmatic nucleus of the hypothalamus (SCN) has been considered the only, master circadian clock which governs all rhythmic changes in physiology and behaviour occurring at a daily timescale. However, evidence accumulating over the last 2 decades questions the supremacy of the SCN (Yoo et al., 2004; Guilding and Piggins, 2007; Paul et al., 2020). With the discovery of intrinsic clock gene expression in several extra-SCN brain nuclei, from the olfactory bulb (Abraham, 2005), through thalamic (Chrobok et al., 2021a), epithalamic (Guilding et al., 2010), hypothalamic (Guilding et al., 2009) and midbrain regions (Chrobok et al., 2021b), all the way to the hindbrain (Kaneko et al., 2009; Chrobok et al., 2020), it is now believed that at least a part of the rhythmic control of homeostasis must be devolved to such local clocks. These circadian timekeeping centres vary in the degree of their autonomy (Guilding and Piggins, 2007; Paul et al., 2020; Chrobok et al., 2021c). An ‘autonomous oscillator’ displays molecular and electrophysiological rhythms that are strongly synchronised amongst its single cells due to highly-functional connectivity within the structure. These coordinated single-cell oscillations are therefore synchronised in phase what results in a robust endogenous rhythmicity at the whole structure level. Similarly, a ‘semi-autonomous oscillator’ shows intrinsic single-cell rhythmicity. Though, as a cause of a sparse interconnectivity, it requires an entraining input from the autonomous clock, synchronising its components to produce high-amplitude rhythms. Last, a “subordinate oscillator” lacks endogenous mechanisms to express intrinsic oscillations. Rather, its rhythmicity reflects and relies on an input from the autonomous or semi-autonomous clock.

Neurons located in the ventral tegmental area (VTA) and substantia nigra pars compacta (SNc) have been suggested to possess such circadian clock properties (Becker-Krail et al., 2022). These are exhibited through circadian variation in clock gene expression, electrical activity, and the concomitant dopamine release in the targeted brain areas. It is still not clear whether these day-night oscillations result from intrinsic properties of dopaminergic neurons, extrinsic sources (such as SCN), or a combination of both. Different evidence points to either of the scenarios, but it is most plausible that dopaminergic system serves as a semi-autonomous oscillator heavily entrained by other circadian clocks. Most importantly, though, these day-to-night variations, despite their origin, influence animal behaviour in a circadian fashion, as dopamine is well positioned to control e.g., the locomotor activity, goal-directed learning, and motivation. Since most rodents are nocturnal, their locomotor activity is significantly higher during dark phase and it decreases during the behaviourally quiescent light phase. In line with this, animal motivation for food seeking, water and food consumption increases during the active phase (Boulos et al., 1980; Ding et al., 2018; Acosta et al., 2020). Similarly, mating behaviour predominately occurs at night, exhibiting clear circadian rhythmicity (Antle et al., 2005). Additionally, drug self-administration has been found to undergo similar daily changes (Roberts et al., 2002; Trujillo et al., 2009). All these daily-regulated behaviours are under control of brain reward system (Cools, 1986; Pfaus et al., 1990; Volkow et al., 2011a; Volkow et al., 2011b; Berridge and Robinson, 2016).

In this perspective, we aim to explore possible sources of daily and circadian rhythmicity in the dopaminergic system. We first focus on abilities of dopaminergic neurons to express molecular and electrophysiological rhythms around 24 h. Then, we describe possible daily patterning of input to the dopaminergic system from other extra-SCN clocks. Finally, we aim to position the midbrain dopaminergic system as one of the hubs in the complex network of brain circadian oscillators.

## Circadian and Daily Rhythms in the Dopaminergic System

The dopaminergic system of the ventral midbrain exhibits circadian variation in the expression of core clock genes, such as *Per1*, *Per2*, *Per3*, *Bmal1* and *Cry* (Bussi et al., 2014; Wang et al., 2019; Koch et al., 2020). Some evidence points out that oscillations in clock genes expression are present within the isolated substantia nigra (Natsubori et al., 2014) and VTA (Landgraf et al., 2016) explant cultures. Other reports, however, using similar methodology, show no or very weak oscillations in the VTA (Abe et al., 2002; Myung et al., 2018) and SNc (Hiler et al., 2008). Thus, even if the VTA/SNc can sustain intrinsic rhythms in clock gene expression, their endogenous timekeeping properties are not overtly robust.

It has also been shown that the electrical activity of VTA neurons changes in the circadian cycle (Luo et al., 2008; Domínguez-López et al., 2014; Fifel et al., 2018). The firing rate of dopaminergic neurons *in vivo* was reported to oscillate in a 12 h cycle (with peaks at the beginning of phases) and the number of spontaneously active dopaminergic neurons to change in a 24 h cycle (with the peak at the end of the dark phase) (Domínguez-López et al., 2014). Additionally, a new population of fast-firing non-dopaminergic and non-GABAergic cells was reported within the VTA, which are active only during the active phase (Luo et al., 2008). The activity of the VTA is also elevated during the night in freely moving mice; in case of the SNc only a non-significant trend was observed (Fifel et al., 2018). Importantly, multiunit recordings were used for data collection, thus distinguishing between cell types contributing to the daily firing alterations was not possible. Nonetheless, night-time increase in firing agrees with reports showing more cFos-immunoreactive dopaminergic cells within the VTA during the night (Baltazar et al., 2013) as well as increased night-time glucose utilization within the SNc (Room and Tielemans, 1989). The disruption in the molecular clock (*Clock* knockout) elevated both firing rate and bursting of dopaminergic neurons *in vivo* (Mcclung et al., 2005). Overall, these data suggest that the electrical activity of dopaminergic neurons undergoes circadian changes, but to what extent this variability depends on changes in their clock genes expression, and to what extent it reflects rhythmic changes in the inputs to the dopaminergic system, remains unclear.

In line with that, studies congruently demonstrate that dopamine concentration is elevated in the striatum during the active phase (Owasoyo et al., 1979; Smith et al., 1992; Paulson and Robinson, 1994; Castañeda et al., 2004; Hampp et al., 2008; Bussi et al., 2014; Ferris et al., 2014; Koch et al., 2020). However, the level and pattern of electrical activity of midbrain dopaminergic neurons does not exhibit as pronounced circadian rhythmicity as it would be expected from the dopamine release rhythm. Thus, it may suggest that other mechanisms of dopamine release regulation may additionally contribute to this phenomenon. Indeed, the expression of genes responsible for synthesis and turnover of dopamine show clear day-night variation. The expression of monoamine oxydase (MAO), which increases dopamine turnover and decay, is lower during the night in both the striatum and VTA (Hampp et al., 2008). Similarly, dopamine transporter (DAT) was shown to be crucial for daily variation in dopamine release (Ferris et al., 2014). Accordingly, the expression of tyrosine hydroxylase (TH), a limiting enzyme in dopamine biosynthesis, is higher during the night in the striatum (Sleipness et al., 2007; Webb et al., 2009; Bussi et al., 2014; Ferris et al., 2014; Koch et al., 2020). Overall, these studies demonstrate that dopamine release increases during night-time, which may be due to daily changes in the activity of dopaminergic neurons and/or the mechanisms that control synaptic dopamine release. In any of these scenarios, the enhanced dopaminergic drive during the dark phase has clear behavioural consequences.

Indeed, it has been shown that the period of locomotor activity in VTA-lesioned rats is significantly decreased (Isobe and Nishino, 2001). Moreover, a lack of D1 dopamine receptor (D1R) in mice attenuates the rate of circadian entrainment (Grippo et al., 2017) and impairs food anticipatory activity (Gallardo et al., 2014). On the other hand, disturbances in the molecular clock influence dopamine-related behaviours, e.g., selective VTA *Clock-*knockouts exhibit increased locomotor activity in the novel environment (Mukherjee et al., 2010). Moreover, drug abuse studies have confirmed that mice lacking *Clock* or *Per2* genes were more strongly rewarded by cocaine administration (Abarca et al., 2002; Mcclung et al., 2005). Conversely, mice with *Per1* knockdown experience no reward after cocaine injection (Abarca et al., 2002). Altogether, these studies reveal intrinsic clock properties of the dopaminergic system that influence daily and circadian rhythms in animal behaviour.

Besides intrinsic circadian timekeeping properties of the VTA, it receives an indirect input from the master clock. It has been shown that the median preoptic nucleus (MPON) serves as a relay between the SCN and the VTA (Luo and Aston-Jones, 2009; Mendoza and Challet, 2014). In return, the VTA directly innervates the master clock, with dopamine able to regulate the rate of photoentrainment of the SCN (Isobe and Nishino, 2001). It has been also shown that this connection plays a key role in brain development, as it synchronises the circadian rhythmicity of the mother and her offspring (Grippo et al., 2020) and, by disruption of food intake timing, can induce overconsumption (Weaver et al., 1992; Viswanathan et al., 1994). Moreover, it is worth mentioning that dopaminergic signalling may be driving the expression of some extra-SCN circadian rhythms, for example in the synthesis of clock protein PERIOD2 in the dorsal striatum (Hood et al., 2010). Importantly, dopaminergic system was shown to be important for maintaining ultradian (∼4 h) locomotor rhythm as either DAT knockout, metamphetamine treatment or chemogenetic activation of dopaminergic neurons lengthened its period (Blum et al., 2014; Bourguignon and Storch, 2017). Moreover, haloperidol had an opposite effect and striatal dopamine levels were shown to correlate strongly with this rhythm. Congruently, dopaminergic system is posed to be important in the control of anticipatory activity rhythms (Steele and Mistlberger, 2015; de Lartigue and McDougle, 2019; Mistlberger, 2020). Thus, we hypothesise that the indirect SCN input to the VTA may synchronise its semi-autonomous rhythms and entrain them to the changing light-dark cycle, but we also highlight the reciprocal character of this connection.

### Circadian Rhythmicity in the Input Pathways to the Dopaminergic System

As mentioned above, the last 2 decades have brought discoveries of several extra-SCN neuronal populations of circadian oscillators with varying degrees of autonomy, which participate in shaping daily changes in physiology, behaviour, and cognitive processes. Unsurprisingly, at least some of these extra-SCN clocks are among the building blocks of the incredibly extensive, monosynaptic inputome of the ventral midbrain dopaminergic neurons (Watabe-Uchida et al., 2012; Ogawa et al., 2014). This may have a twofold effect on the functioning of the dopaminergic system.

**FIGURE 1:**
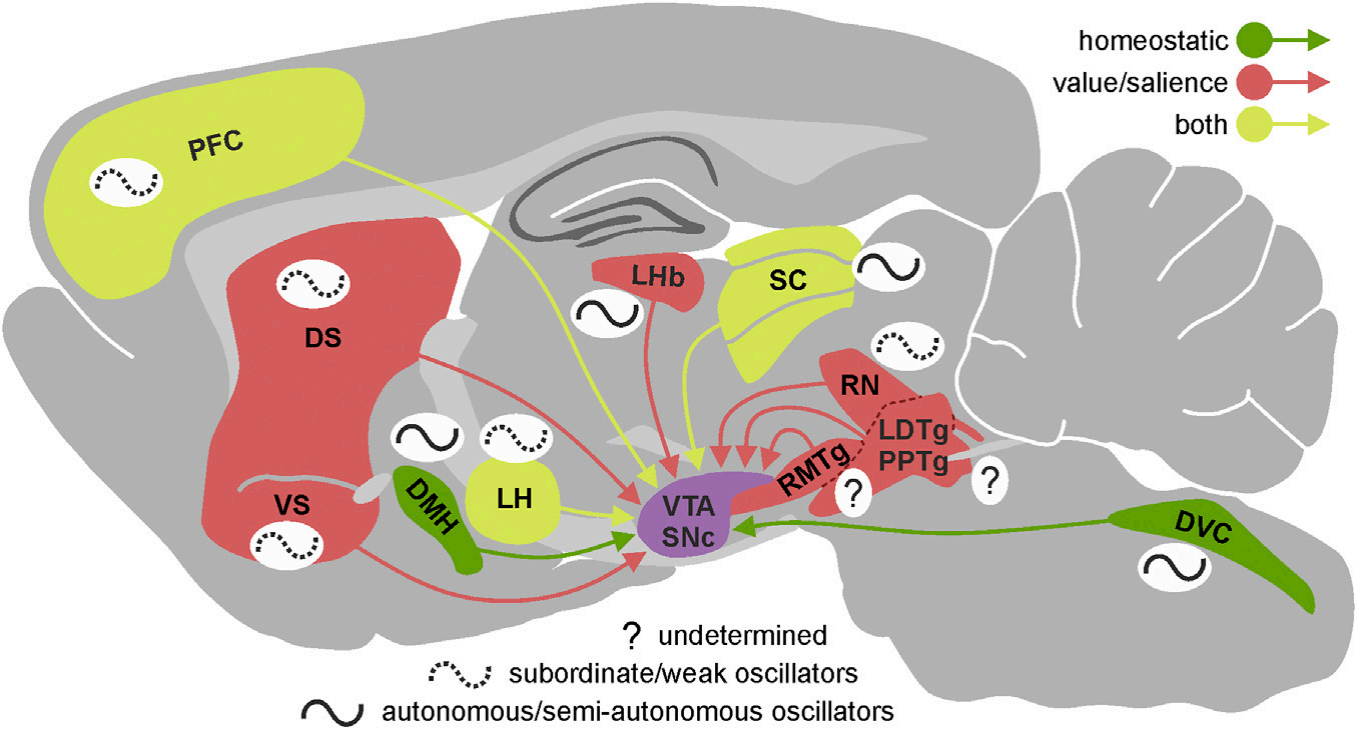
Circadian properties of the VTA/SNc inputs. Presence and autonomy of circadian rhythmicity in the most important brain regions innervating VTA/SNc presented on the sagittal section of rat brain. Brain regions are divided according to the type of information they provide dopaminergic system with—green: homeostatic, red: value/salience, yellow: both. The schematic wave next to each brain region represents its circadian rhythmicity. Solid line indicates high level of autonomy (autonomous/semi-autonomous oscillator) and dotted line indicates low level of autonomy (subordinate/weak oscillator) while question mark shows that we currently lack the knowledge about circadian properties of a given brain region. VTA, ventral tegmental area; SNc, substantia nigra pars compacta; DS, dorsal striatum; VS, ventral striatum; LHb, lateral habenula; RMTg, rostromedial tegmental nucleus; LDTg, laterodorsal tegmental nucleus; PPTg, pedunculopontine tegmental nucleus; RN, raphe nucleus; SC, superior colliculus; LH, lateral hypothalamus; DMH, dorsomedial hypothalamic nucleus; DVC, dorsal vagal complex; PFC, prefrontal cortex.

First, the circadian rhythmicity in the electrical activity present in these input structures may translate into changes of similar, hourly dynamics in the level of dopaminergic neuron firing and concomitant dopamine release in target brain regions as described above. Indeed, such circadian changes have been observed in multiple brain nuclei innervating dopaminergic neurons; with these neuronal centres being the source of those inputs spread throughout the whole cerebrum and carrying information of various kinds. Some of them are involved in maintaining homeostasis of the organism (e.g., dorsomedial hypothalamus; LH, lateral hypothalamus; NTS, nucleus of the solitary tract; Figure 1) (Kirouac and Ciriello, 1997; Leinninger et al., 2009; Mejías-Aponte et al., 2009; Leinninger et al., 2011; Tyree and de Lecea, 2017; Alhadeff et al., 2019) processing of sensory information (e.g., SC, superior colliculus) (Peter and Kevin, 2006; Redgrave et al., 2010; Chrobok et al., 2021b; Pradel et al., 2021), controlling general states of arousal (e.g., LH; tuberomammillary nucleus) (Azeez et al., 2018; Heiss et al., 2018), and carrying out cognitive processes (e.g., PFC, prefrontal cortex; Figure 1) (Carr and Sesack, 2000; Chun et al., 2015; Calabrese et al., 2016; Chen et al., 2016; Seney et al., 2019).

Second, it has been shown that the motivational aspects of stimuli perceived by the animal are encoded in the electrical activity of dopaminergic neurons, based on information from other regions of the brain, which often carry a partially or fully encoded value or salience of the stimulus (Tian et al., 2016; de Jong et al., 2019). Circadian changes in electrophysiological properties of the elements building the internal neural networks of such structures may affect the information passed on to the dopaminergic neurons. In fact, daily changes in gene expression (including clock genes) and/or electrical activity have been observed in many structures and neuronal pathways involved in encoding the motivational and salient features of stimuli, including the medial prefrontal cortex (Chun et al., 2015; Calabrese et al., 2016), dorsal and ventral striatum (Masubuchi et al., 2000; Ángeles-Castellanos et al., 2007; Wang et al., 2019), lateral habenula–rostromedial tegmental nucleus pathway (LHb-RMTg) (Zhao and Rusak, 2005; Guilding et al., 2010; Sakhi et al., 2014; Baño-Otálora and Piggins, 2017), LH (Marston et al., 2008; Azeez et al., 2018), dorsal vagal complex (Kaneko et al., 2009; Chrobok et al., 2020; Chrobok et al., 2021d; Chrobok et al., 2021e; Chrobok et al., 2021f) and dorsal raphe nucleus (Abe et al., 2002; Paul et al., 2020) (Figure 1). It is noteworthy, that in case of some important inputs to the dopaminergic system we lack even basic information about their potential circadian rhythmicity (e.g., pedunculopontine tegmental nucleus, laterodorsal tegmental nucleus, RMTg).

Therefore, it seems that the circadian changes in the electrophysiological properties of the structures innervating dopaminergic neurons may translate into long-scale (occurring in hours) changes in the basal activity of the dopaminergic system, as well as changes in the short-scale (occurring in milliseconds or seconds) phasic responses of DA neurons that encode value and/or salience of the perceived stimuli. Likewise, circadian changes in long-scale and short-scale dopaminergic activity should be apparent at the level of the animal’s behavioural states and behaviours.

Importantly, daily changes in the dopaminergic system happening on the long timescale seem to depend predominantly on the intrinsic brain mechanisms (i.e., clock genes expression along with multi-clock network activity); notably, these are shaped by the long-lasting environmental conditions (i.e., light/dark cycle) (Mendoza and Challet, 2014; Becker-Krail et al., 2022). On the other hand, the dopaminergic system functioning on the short timescale is extremely sensitive to brief cues appearing in the environment (e.g., reward-associated stimuli) (Schultz, 2016). Additionally, these short responses may also be modulated by the current electrophysiological state of dopaminergic neurons, which is a manifestation of long timescale circadian oscillations.

## Open Questions and Further Research Directions

### Circadian Changes in Fast and Slow Dynamics of the Dopaminergic System

The pattern of electrical activity that a dopaminergic neuron exhibits at a given moment can be positioned on a spectrum ranging between two extremes: tonic, pacemaker-like firing of action potentials and, on the other end, generation of action potentials in purely bursting manner (Grace and Bunney, 1984). These two firing modes are controlled by different input signals and their contribution to the basal activity of dopaminergic neurons differs amongst behavioural states (Robinson et al., 2002; Lodge and Grace, 2006; Geisler et al., 2007; Tsai et al., 2009; Zweifel et al., 2009; Watabe-Uchida et al., 2012). Relatively slow changes in dopaminergic neuron excitability occurring on a scale much longer than seconds or minutes may affect the functioning of the dopaminergic systems in two ways. First, by alerting the propensity to generate action potential bursts and thus setting the ratio of tonic to burst firing, it determines the basal dopamine release in target structures. This in turn affects some aspects of the general behavioural state of an animal (e.g., level of arousal, behavioural invigoration, tendency to effort, and energy expenditure) (Parsons and Justice, 1993; Floresco et al., 2003; Cagniard et al., 2006; Niv et al., 2007; Wang et al., 2021). Second, changes in excitability may affect how short synaptic events translate into momentary changes in dopaminergic neuron firing and thus into phasic increases and/or decreases in dopamine release. Such brief changes in dopamine release, usually lasting less than a second, encode the value and/or salience of stimuli perceived by the animal at a given moment and are the basis of many cognitive processes such as experience-based learning, decision-making, and directing attention (Schultz et al., 1979; Stuber et al., 2005; Day et al., 2007; Stuber et al., 2008; Sunsay and Rebec, 2008; Dreyer et al., 2010; Jastrzebska et al., 2016; Stojakovic et al., 2018; Budygin et al., 2020). Although slow and fast dynamic changes in the dopaminergic system have been extensively described in the literature, the open question remains and how they are affected by the circadian clock.

Our current knowledge sheds light on the contribution of the circadian clock machinery to some of the slow changes observed in the activity of the dopaminergic system. Mostly, it describes the diurnal variation in the clock gene expression, overall electrical activity, concomitant dopamine release, as well as daily changes in the animal behaviour. These observations are consistent with the fact that in many neuronal populations circadian clock gene expression is accompanied, often causally, by changes in the expression of genes determining the membrane properties, neuron responsiveness to neurotransmitters, and the effectiveness of further signal transmission (Paul et al., 2020). In fact, it has been shown that the expression of genes coding some potassium channels, subunits of glutamate and GABA receptors, and proteins related to dopamine release in dopaminergic neurons depend on molecular clock (Mcclung et al., 2005). Such changes in the electrophysiological properties of neurons should directly translate into circadian alterations of both slow and fast dynamic phenomena occurring in the dopaminergic system and related behaviours. Still, there are many gaping holes in our current knowledge on the subject. We still know very little about whether and how the circadian, slow changes in the properties of neurons affect the fast processes taking place in the dopaminergic system, such as encoding value or salience stimuli. Moreover, as it will be discussed in more detail in the next section, it is likely that the circadian changes in VTA/SNc input structures involved in the calculation of the reward prediction error are also reflected at the level of dopaminergic system.

### Circadian Changes in Dopaminergic System Upstream and Downstream Structures

As already described above, dopaminergic system receives inputs from multiple regions distributed throughout the whole brain. Since presumably both the baseline activity and responsiveness to environmental cues of these inputs alter across day/night cycle, magnitude of dopaminergic system excitation/inhibition evoked by those inputs should be affected as well. More precisely, it is important to establish how the parameters (such as amplitude, duration, latency, or even polarity) of responses of dopaminergic neurons to value/salience information provided by different inputs (e.g., PFC, SC, LHb, LH; Figure 1) change at different times of a daily cycle. Assuming that the dopaminergic system acts as a hub that computes and sums incoming information, and the circadian oscillation of VTA/SNc inputs change the weight of information they carry, the output information from the dopaminergic system should be appropriately adjusted. The question posed above needs an urgent answer because previous experiments on the responses of dopaminergic neurons to manipulations of its inputs were not designed to measure differences between light and dark phases of the circadian cycle. Additionally, it should be delineated to what extent the dopaminergic signalling is affected by its endogenous circadian rhythmicity (and concomitant changes in electrophysiological properties), or by external circadian rhythmicity within individual VTA/SNc input structures. Notably, almost none of the inputs that reach dopaminergic system are homogeneous biochemically and structurally. Thus, understanding how daily oscillations within particular brain region as a whole might be insufficient to explain its real impact on the neuronal activity of the dopaminergic system across 24 h; different cellular populations within one brain region which often fulfil different functions and convey different information might undergo dissimilar circadian changes.

It is expected that both basal dopamine levels as well as value/salience encoding should differ in active and inactive phases as they pose different behavioural challenges to animals. For example, the impact of the ambient light level and environmental cues on the activity of the dopaminergic system during both phases should differ; indeed, it was shown that the activity of superficial layers of the SC is lower during the night (Chrobok et al., 2021b). The same should apply for the homeostatic information provided by various hypothalamic regions as well as the NTS (Chrobok et al., 2021d; Chrobok et al., 2021f), as the food intake along with the motivational drive to eat varies strongly between active and inactive phases. Some of these hypothalamic structures also regulate the general arousal state so the daily variation in their activity is also anticipated to affect the activity of the dopaminergic system to a great extent. The way dopaminergic neurons respond to aversive and threatening stimuli in active and inactive phase most likely differ as distinct rodents’ predators lurk at different times of a day. Moreover, alertness to such information is different while the animals are resting. For that reason, daily changes in the LHb and RMTg responsiveness (and baseline activity) should not come as a surprise. Indeed, it was shown that the activity of the LHb varies across 24 h cycle (Baño-Otálora and Piggins, 2017). As a result of the multi-input circadian oscillations, the change in the activity of the dopaminergic system in both short and long timescales should translate into daily dopamine release alterations in the target brain areas, such as the striatum. This relation, however, does not have to be linear as various daily changing factors at the level of the striatum itself might influence the dopamine release (e.g., activity of cortical inputs or dopamine level controlling enzymes expression).

## Concluding Remarks

The current development in understanding central circadian timing mechanisms, shifting from the uni-clock concept of the omnipotent SCN towards the more complex multi-clock theory, provides new insights in the regulation of discrete neuronal systems. The dopaminergic system serves as an interesting example. The combination of intrinsic circadian timekeeping in the dopaminergic system itself with the circadian fluctuation of multiple inputs to the VTA/SNc from local brain clocks (exhibiting a range of autonomy from the SCN), ensures much more flexibility than a single, circadian input from the master clock. This assumption can be further strengthened by the fact, that these different “ticking” inputs are entrained by different environmental stimuli. With the SCN being a light-entrainable oscillator poorly entrained by feeding, the input from other extra-SCN clocks may provide information e.g., on the circadian patterning of food intake, valuable for the dopaminergic system functioning. Thus, considering the dopaminergic system as a hub in the intricate network of circadian oscillators, drawn in our perspective article, offers novel research opportunities in both reward and circadian neuroscience.

## Data Availability

The original contributions presented in the study are included in the article/Supplementary Material, further inquiries can be directed to the corresponding authors.
